# Immersive virtual reality psychotherapy for children and adolescents—A systematic review and meta-analysis

**DOI:** 10.1016/j.invent.2026.100920

**Published:** 2026-02-13

**Authors:** Steffen Massanneck, Lennart Seizer, Nadine N. Schmitt, Anja Pascher, Johanna Löchner

**Affiliations:** a Clinical Psychology and Psychotherapy for Children and Adolescents, Friedrich-Alexander University Erlangen-Nürnberg, Erlangen, Germany; b Department of Child and Adolescent Psychiatry, Psychosomatics and Psychotherapy, University Hospital Tübingen, Tübingen, Germany; c German Center for Mental Health (DZPG), Site Tübingen, Germany

**Keywords:** Virtual reality, Metaverse, Systematic review, Adolescence, Mental health, Meta-analysis

## Abstract

Although virtual reality has been used in psychotherapy since the 1990s, research and clinical practice have focused strongly on its application in exposure therapy. Over the last decade, the emergence of a new generation of virtual reality devices has led to a surge in virtual reality applications, which are also gaining traction in other areas of mental health care. The use of virtual reality in children and adolescent psychotherapy is especially interesting because it provides engaging and fun opportunities to strengthen therapeutic success. Despite these emerging needs, most systematic reviews focus not on high-immersive virtual reality applications but instead include low-immersive virtual reality applications, which may influence the results. This review fills the gap by providing a systematic review and meta-analysis of high-immersive virtual reality applications for child and adolescent psychotherapy. A total of nine eligible randomized controlled trials were included. The meta-analysis highlights that virtual reality applications can significantly enhance therapeutic success (Hedge’s *g* = −0.25, 95% CI [−0.37, −0.13]). During the extensive literature search, several research gaps were identified. Future research should concern itself with additional RCTs on mental health conditions that are currently lacking, such as Learning Disorders, Obsessive-Compulsive Disorder (OCD), Depression, and others. Initially, we also searched for augmented reality applications, but our literature search revealed no suitable studies.

## Highlights

• VR applications are comparably effective as active treatments, such as standard CBT.
• VR intervention was more beneficial in 52 of 67 cases (fewer symptoms/better skills).
• Realistic avatars were associated with more beneficial VR treatment effects.
• High interactivity had a positive effect on the patients’ mental health measures.

## 1 Introduction

Mental Health disorders pose a huge threat to children and adolescents, as of 2019, 8.8% of children and adolescents have been diagnosed with various mental health disorders (Piao et al., 2022). Further, about half of all mental disorders have their onset prior to the age of 18 (Solmi et al., 2022). To treat these conditions, child and adolescent psychotherapists face multiple unique challenges compared to their colleagues. Children and adolescents in psychotherapy face developmental challenges (Löchner and Schuller, 2023) and have special needs, such as a desire for engaging and playful forms of therapy (Bhide and Chakraborty, 2020). Emerging immersive technologies such as Virtual Reality (VR) and Augmented Reality (AR) offer new possibilities for providing novel, engaging, playful, and fun forms of psychotherapeutic sessions (Lindner, 2021, Alt et al., 2024, Tomczyk and Gottschalk, 2024).

### 1.1 Extended reality

Extended Reality (XR) is defined by Venkatesan et al. (2021) as *”all real and virtual combined environments generated using computers and wearables, such as VR, AR, and MR technologies. XR includes the entire spectrum of the reality-virtuality continuum from absolute reality to utter virtuality”*. Virtual reality (VR) is situated at the *utter virtuality* end of the spectrum, and augmented reality (AR) at the *absolute reality* end, with mixed reality (MR) falling between the two (Milgram and Kishino, 1994, Venkatesan et al., 2021). VR can be defined as *“... immersive technologies to simulate interactive virtual environments or virtual worlds with which users become subjectively involved and in which they feel physically present”* (Wohlgenannt et al., 2020). For creating this interactive virtual environment, three-dimensional images, sounds, and sensoric feedback, such as haptic feedback, can be integrated (Smutny et al., 2019). The content can be displayed using output devices such as computer displays, projection walls, and head-mounted displays (HMDs), also known as VR headsets (Huang et al., 2023). HMDs can provide a highly immersive environment by using two lenses to create three-dimensional stereoscopic images through the display of one two-dimensional image per eye (Venkatesan et al., 2021). Users of virtual environments can interact using input devices such as head tracking, eye tracking, body tracking devices, controllers, voice input, and common computer input devices (Song and Qin, 2022, Venkatesan et al., 2021).

According to Rutkowski et al. (2021), VR devices (and corresponding software) can be classified into two categories: (1) non-immersive VR and (2) immersive VR. The first category, non-immersive VR, refers to applications that are displayed on a computer screen; therefore, the external real environment remains visible to the user. While immersive VR describes applications in which the user’s interactions in the virtual environment cannot be differentiated from those in the real environment and different senses, such as sight, hearing, touch, or smell, can be replicated using hardware such as a VR headset (Musa et al., 2022, Rutkowski et al., 2021). In contrast to VR, AR allows one to view the physical world, which is enriched by virtual objects displayed onto it (Silva et al., 2018). The user wears an HMD with a transparent display, allowing them to see both the enriched physical world and virtual objects (Silva et al., 2018).

To date, psychotherapeutic research and clinical practice have primarily focused on the use of VR in exposure therapy, also known as “virtual reality exposure therapy” (VRET). A systematic literature review emphasizes this (Valmaggia et al., 2016), as they investigated the use of VR for treating mental disorders of adult patients and mainly found studies connected to treating phobias, such as agoraphobia, social phobias, and fear of flying. In addition, effects for the diagnosis and treatment of people with psychosis autism spectrum disorders, and attention deficit hyperactivity disorder were investigated (Lan et al., 2023). Further studies are based on addiction and eating disorders and — however a limited number — on generalized anxiety disorder and obsessive-compulsive disorder (Emmelkamp and Meyerbröker, 2021).

In recent years, VR research has emerged to investigate social VR applications, often referred to as the metaverse, which further blur the boundaries between physical and virtual worlds (Dwivedi et al., 2022). A metaverse is a new iteration of the internet where VR headsets, blockchain, and avatars connect the physical and virtual world in an integrated, immersive ecosystem (Dwivedi et al., 2022).

### 1.2 Previous reviews

Previous reviews, like Ridout et al., 2021, Varma et al., 2022, Wiebe et al., 2022, have investigated the use of immersive technologies and virtual environments in child and adolescent psychotherapy. However, there are several limitations to these and other reviews. Due to the rapid technical progress in VR headsets over the past few years, devices with higher resolutions, enhanced interaction capabilities, wireless functionality, and customizable avatars have been released (Radianti et al., 2020, Bondarenko et al., 2024, Meta, 2025). In consequence, some released reviews no longer capture the technological state of the art. Others only used one database or a very narrow search term, such as only including VR but not AR or similar immersive technologies, or only using *children* in the search term without considering synonyms. Some other reviews included non-immersive VR, such as traditional computer games, in their review, thereby distorting the validity of the results’ interpretation for high-immersive applications. The majority of reviews also included Cave Automatic Virtual Environments (CAVE) in their review. CAVE can be described as a dedicated cubic room equipped with multiple displays on each wall, which simulates a virtual world (Cruz-Neira et al., 1993). This is a problem because one barrier to incorporating VR in clinical research and practice is the corresponding budget of these systems and maintenance costs (Garrett et al., 2018). Additionally, due to the assumed heterogeneity of previous studies, which involved treating different mental disorders, no meta-analysis was conducted in prior reviews.

### 1.3 Current study

While systematic reviews have mapped out the landscape of immersive-technology interventions in youth psychotherapy, existing meta-analyses remain confined to single diagnostic categories (e.g., phobias, PTSD). No work to date has synthesized effect sizes across the full spectrum of child and adolescent clinical applications. To address this gap, we undertook a comprehensive meta-analysis that (1) pools overall efficacy estimates for VR/AR–based treatments, (2) quantifies between-study heterogeneity, and (3) separately contrasts outcomes against active (e.g., CBT, EMDR, role-play) versus passive (e.g., waitlist, no-treatment) control conditions.

Moreover, we focus on full-immersive VR and augmented reality (AR) applications, assuming higher effectiveness due to increased engagement and immersion (Bell et al., 2020). Additionally, we aim to prioritize affordable VR and AR devices that are viable for all psychotherapists and clinics, including solo practitioners, smaller clinics, and those in lower-GDP countries, as one barrier to a higher reach has been the high costs of equipment (Bell et al., 2020). Therefore, we will pay attention to VR HMDs, which require only limited separate infrastructure, are more affordable than CAVE systems and are in a price range suitable for solo practitioners, in consultation with licensed therapists.

Because some studies have shown initial indications of the potential effectiveness of remote VR psychotherapy sessions (Turan Akdag et al., 2023, Massanneck et al., 2024), we aim to investigate further whether remote trials have been conducted and demonstrate similar effectiveness compared to in-person therapy.

Expert interviews conducted by Ong et al. (2024) also indicated that realistic virtual avatars and interactivity are important for the usefulness of VR telepsychotherapy applications; therefore, we want to investigate these characteristics as moderators of treatment efficacy. Another factor we want to quantify is usability, because a high usability is crucial for the acceptance of new therapeutic forms, such as telepsychotherapy (von Below et al., 2023).

## 2 Methods

The current study utilizes the Preferred Reporting Items for Systematic Reviews and Meta-Analyses flow diagram (PRISMA) (Page et al., 2021).

### 2.1 Study selection

We used the PICOS scheme (Participants, Interventions, Comparisons, Outcomes, Study Designs) (Hoogendam et al., 2012) as the foundation for defining our study inclusion criteria. Studies were included if their participants were not older than 18 years (P), who received a high-immersive VR or AR intervention through HMDs related to psychopathological symptoms (I), compared to active or passive control groups , e.g. psychotherapy or waitlist control group. Studies including participants with psychopharmacological treatment as a separate control group were excluded to highlight the comparison of VR /AR (C). Outcome (O) was defined as the change in symptom severity as a result of the psychological intervention. Thereby, different measures were used based on the underlying disorder. Because immersive VR in child and adolescent psychotherapy is an emerging and still niche research area, we did not limit the type of mental health conditions to include as many studies as possible. This approach was chosen to maximize coverage of the available evidence and to characterize the breadth of current applications. However, we acknowledge that broader inclusion reduces disorder-specific interpretability and increases heterogeneity in outcomes. We only considered Randomized Controlled Trials (RCT) with a pre-post-design and reported pre- and post-mean and standard deviation values to be able to calculate effect sizes for the meta-analyses (S). If no values were reported, the corresponding authors were contacted. Furthermore, we only considered studies written in English and German.

### 2.2 Search strategy

**Fig. 1:**
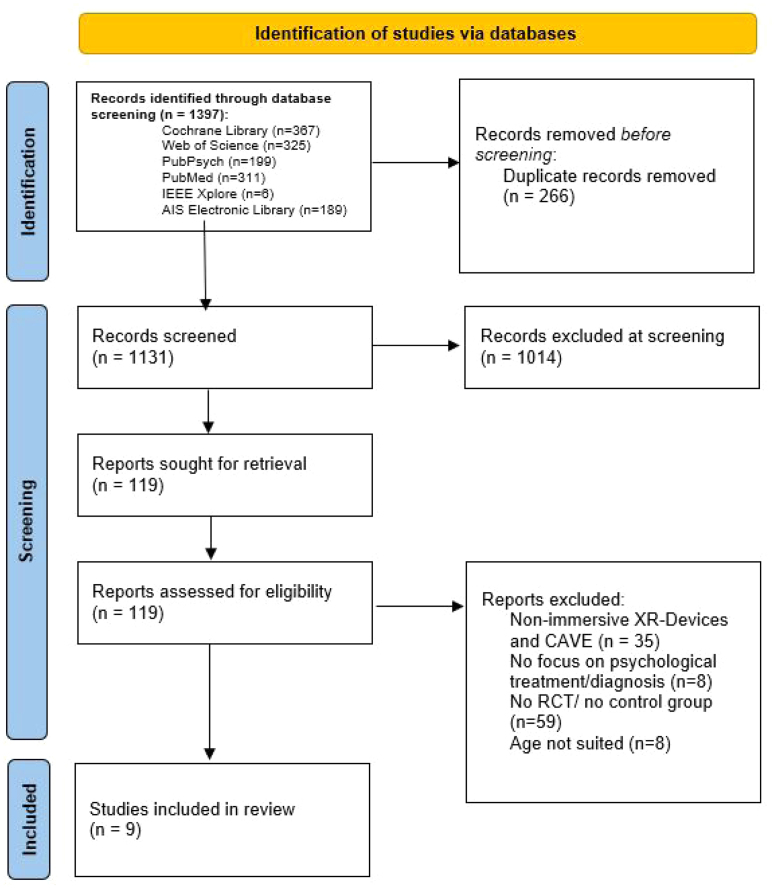
PRISMA-Flowchart.

A literature search was conducted in the following four databases — PubMed, Web of Science, PubPsych, and Cochrane Library — in October 2024 to identify relevant studies. We also searched IEEE Xplore and the AIS Electronic Library in January 2026 to potentially expand the included studies to those that are technically feasible. Fig. 1 shows a PRISMA-Chart for the Selection Process. We included RCTs from 2016 to 8th October 2024. The beginning of this period (2016) was chosen, as it was the release year of the consumer version of the Oculus Rift Headset, which was one of the first headsets to overcome the technical limitations of previous VR headsets, such as narrow fields of view, low frame rates, and suboptimal latencies (Farahani et al., 2016). To find as many relevant RCTs as possible, we used the search term in the Appendix to filter in title and abstract.

We also attempted to search for ongoing trials on ClinicalTrials.gov to include unfinished trials; however, none were found that provided sufficient preliminary results to calculate the effect size. In total, 1397 references were found. After removing the duplicates (k = 266), the titles and abstracts of these remaining references were screened according to the eligibility criteria. A total of 119 references were retrieved and assessed for eligibility through a full-text screening. In this stage, (k = 110) studies were excluded, due to use of non-low-cost, like CAVE, or non-immersive XR devices, like ordinary computer displays (k = 35), missing focus on a treatment suitable for psychological counseling (k = 8) this included Studies focusing on painallevation during operations like Chang et al. (2022), because there are not mainly suitable for psychologists instead mainly for medical areas outside of psychology, non-suitable study design, like no RCT or missing control group (k = 59), age restriction violated (k = 8). A total of nine studies were included in the systematic review and meta-analysis.

### 2.3 Data analysis

All analyses were conducted using *R* 4.3.2 (R. Core Team, 2025) with the *metafor* package for meta-analyses (Viechtbauer, 2010). Source code and output for all analyses are available at the following link https://osf.io/9hmev/?view_only=6f9ba1abd55a41df864b6ebd52b9ead0. Because multiple outcome measures were included in each study, and some studies had multiple control groups, a four-level meta-analysis was conducted across all studies. Our four-level meta-analysis therefore accounts for Sampling variance (Level 1), between-outcome variance (Level 2), between-control group type variance (Level 3), and between-studies variance (Level 4). This was followed by two three-level meta-analyses, consisting of Sampling variance (Level 1), between-outcome variance (Level 2), and between-studies variance (Level 3), to differentiate the effect sizes more accurately between different treatment approaches. One compares the effect of the virtual reality intervention group with passive control groups, such as a waitlist control group or no treatment, and another compares the effect of the VR intervention group with active control groups, such as standard care using cognitive-behavioral therapy (CBT). Because most studies were conducted in different countries resulting in populations with heterogeneous cultural background in addition to targeted disorder, for example, children with acrophobia (Azimisefat et al., 2022) and children with ADHD (Bioulac et al., 2020), we expected no relevant sample overlap. Most studies reported measures for different mental health conditions; therefore, a random effects model was used, as it was unlikely that the studies shared a single true effect size.

To compare the studies’ outcomes, Hedges’ *g* (Hedges, 1981) and corresponding 95% confidence intervals were calculated. Between-group comparisons of pre-post change scores were calculated using pooled pre-test standard deviations with the small sample bias correction, as some samples were small, e.g., Porras-Garcia (2020) (n=17), following the approach outlined by Morris (2008). The parameter $d_{pcc2}$ was used with the given formulas

$d_{pcc2}=c_{p}\left[\frac{\left(\left(M_{post,T}-M_{pre,T}\right)-\left(M_{post,C}-M_{pre,C}\right)\right)}{{\mathrm{SD}}_{pre}}\right],{\mathrm{SD}}_{pre}=\sqrt{\frac{\left(n_{T}-1\right){\mathrm{SD}}_{pre,T}^{2}+\left(n_{C}-1\right){\mathrm{SD}}_{pre,C}^{2}}{n_{T}+n_{C}-2}},c_{p}=1-\frac{3}{4\left(n_{T}+n_{C}-2\right)-1}$The calculation of Hedges’ *g* according to Morris (2008) relies on the correlation between pre- and post-measures, because no correlations was reported, we assumpted r = .50. A sensitivity analysis was conducted to investigate if assuming different values for r, changes the results, with values of r =. 40 and r = .60. In case of RCTs with three or more arms, for each arm an additional outcome was investigated. E.g., Alsem et al. (2023) included two additional arms (“Usual Care” and “Roleplay”). Two comparisons per outcome were created: VR vs. Usual Care and VR vs. Roleplay, and these were treated as distinct outcomes. To account for this, as stated above, a four-level meta-analysis was conducted. In total 67 outcomes were compared. Because the majority (k = 46) were negatively coded (i.e., lower values indicating a positive effect), we inverted the effect sizes of the other outcomes by multiplying them by −1 (k = 21). For interpreting effect sizes, Cohen’s rule of thumb (small 0.20–0.49, medium 0.50–0.79, large higher than 0.80) was used (Cohen, 1988).

The heterogeneity between trials was estimated by Q- and $I^{2}$-statistics (Higgins and Thompson, 2002). To calculate Q, the multivariate function of the metafor-package was used. To calculate the $I^{2}$-statistics, we used the formula (Nakagawa and Santos, 2012) gave for the four-level meta-analyses. For the two three-level-meta-analyses we used the dmetar-package, which implements the formula of Cheung (2014). For interpreting the $I^{2}$-statistics, we used the recommendations of the Cochrane Handbook for Systematic Review of Interventions (Higgins et al., 2024): 0% to 40%: heterogeneity might not be important; 30% to 60%: may represent moderate heterogeneity; 50%–90% may represent substantial heterogeneity; 75% to 100%: considerable heterogeneity.

### 2.4 Coding

Four of the co-authors extracted and coded independently data from each of the nine studies twice. All discrepancies were resolved through discussions by the researchers. The means and standard deviations of the pre- and post-measurements were extracted from the studies to calculate the effect sizes of the studies’ outcomes. Because only three studies measured follow-up outcomes, only pre- and post-outcomes were considered. Additionally, possible moderators were coded, including the use of avatars, the degree of estimated usability, and the degree of interactivity. Coders were blinded and independent from each other. These moderators were coded as follows. Low interactivity indicates that no movement is possible in the virtual environment. The user stays static in the virtual room. Examples for this are 360-degree videos. Another characteristic of a low-interactivity application is the absence of interactable objects in the virtual environment. In contrast, high interactivity means that, e.g., interactions with virtual objects and/or movements in the virtual environment are possible. Characteristics of a low-usability application include no introduction session to explain the VR controls, no tooltips, and no other help within the application. By contrast, a high usability application should provide an introduction to VR controls, as it is likely a new technology for most participants, with tooltips or other help within the application. A middle-usability is characterized by some parts of high usability but not all. For the avatar moderator, there were three possible values: no, low quality, and high quality. No indicates a total absence of virtual avatars in the application. Low quality is characterized by the appearance of non-realistic avatars, e.g., low resolution, fantasy-like, and no or only limited facial expressions. High quality is characterized by high-resolution avatars with high-quality facial expressions.

The interrater agreement was high, ranging between 88.9% and 100% for all variables. Additionally, Cohen’s κ was calculated for the Interrater agreement of moderator coding of the variables Avatar, Interactivity, and Usability. For Avatar and Usability, we obtained a Value of 1; for Interactivity, 0.77 was measured. All values can be interpreted as substantial to perfect (Landis and Koch, 1977) All conflicts could be solved through discussion between the coders.

### 2.5 Risk of bias

To evaluate the study quality Cochranes Risk of Bias 2 (RoB) was used. The plots were created by using the R package *robvis* (McGuinness and Higgins, 2020).

## 3 Results

### 3.1 Study characteristics

Table 1 describes the study characteristics. Nine eligible studies, involving 929 children and adolescents, were identified, yielding 67 effect sizes. Most studies tended to be small, with a sample size ranging from 17 to 378 (Mean = 103.2). The ages of the participants ranged between 6 and 18 years. Only eight studies reported gender ratios and mean age. In total, 507 out of 929 were male (54.57%). The mean age of tested children was 13.28 based on the eight studies that reported data. Two studies were conducted in Iran and one in the Netherlands, France, Denmark, the United States, Norway, Spain, and China. Three studies focused on Attention deficit hyperactivity disorder (ADHD). One study each investigated aggressive behavior, acrophobia and anxiety, drug refusal skills, improving sexual risk prevention behavior, public speaking anxiety, and eating disorders. In seven out of nine studies, a specific diagnosis or scores indicating clinical severity were required to participate. Only Hadley et al. (2019) and Guldager et al. (2022) did not specify requirements for diagnosis or certain scores and included all students who wished to participate. Three studies did not specify the type of VR device (Bioulac et al., 2020, Hadley et al., 2019, Tabrizi et al., 2020). Two were using Oculus Rift devices (Alsem et al., 2023, Azimisefat et al., 2022), three Oculus Quest devices (Guldager et al., 2022, Kahlon et al., 2024, Wong et al., 2024), and one used HTC-VIVE (Porras-Garcia, 2020). No Study used an AR device.

**Table 1:** Study characteristics and demographics.

Study	Country	VR device	Target population	Age range	Sample size	Gender (boys/girls)
Alsem et al. 2023	Netherlands	Oculus Rift S	Aggressive behavior problems	8–13 years	115	115/0
Azimisefat et al. 2022	Iran	Oculus Rift DK2	Acrophobia	16–18 years	45	0/45
Bioulac et al. 2020	France	Unspecified	ADHD	7–11 years	51	41/10
Guldager et al. 2022	Denmark	Oculus Quest	Improve Alcohol Refusal skills	15–18 years	378	186/192
Hadley et al. 2019	United States	Unspecified	Prevention of risk behavior	12–15 years	85	44/41
Kahlon et al. 2023	Norway	Oculus Quest	Public speaking anxiety	13–16 years	100	16/84
Porras-Garcia et al. 2020	Spain	HTC-VIVE	Anorexia nervosa	14–18 years	17	Not reported
Tabrizi et al. 2020	Iran	VR HMD (unspecified)	Memory of ADHD	7–12 years	48	32/16
Wong et al. 2024	China	Oculus Quest 2	Empowering Social Growth for Children with ADHD	6–12	90	73/17

### 3.2 Interventions

Each study differs in terms of which VR application was used, or developed, the age range of the included children, the control conditions, the measured mental health outcome, and the length of the intervention. Therefore, in the following section, we provide a detailed description of all nine interventions. A detailed description of the intervention characteristics is provided in Table 2.

Seven studies aimed at reducing symptoms or improving skills concerning existing diagnoses or behaviors. Two studies aimed at improving preventive Risk Behavior, e.g., through improving drug refusal skills or self-efficacy in HIV Prevention (Guldager et al., 2022, Hadley et al., 2019).

In five studies (Alsem et al., 2023, Bioulac et al., 2020, Kahlon et al., 2024, Tabrizi et al., 2020, Wong et al., 2024), a virtual classroom environment was created for the participants to interact in. Virtual avatars, either for the participants themself or for simulating other people in the virtual environments, were adopted by seven studies. Extensive interactive elements inside the virtual environments were offered by five studies (Alsem et al., 2023, Bioulac et al., 2020, Guldager et al., 2022, Hadley et al., 2019, Wong et al., 2024). These interactive elements encompassed, among other elements, climbing, playing games in the schoolyard, and making choices for social situations.

The length of the VR interventions ranges between a one-time treatment (Guldager et al., 2022) and twelve weeks (Wong et al., 2024). In total one (Guldager et al., 2022) to 15 (Kahlon et al., 2024) sessions with a reported duration range of three minutes (Tabrizi et al., 2020) to two hours (Hadley et al., 2019) were executed. Three studies, such as Alsem et al., 2023, Kahlon et al., 2024, Wong et al., 2024 involved parents or teachers.

Only one study was conducted in a remote setting (Kahlon et al., 2024).

The following sections will provide a more detailed description of all nine interventions. The interventions were clustered into suitable categories. Even though (Wong et al., 2024) investigated Children with ADHD, they focused on improving social interactions. Therefore, the study was not clustered into the ADHD studies but into the social skills category.

**Table 2:** Summary of primary aims, outcome measures and control groups of studies.

Study	Primary aims	Control Groups	Outcome measures	Intervention duration & frequency	Remote	Interactivity	Avatars	Usability
Alsem et al. 2023	Reduce aggressive behavior	Roleplay Intervention Care-as-usual	Aggression Frequency Scales for Child, Parents, Teacher	10 sessions in 10 weeks [60 min]	No	High	Low-quality	High
Azimisefat et al. 2022	Reduce symptoms of acrophobia	Eye Movement Desensitization and Reprocessing (EMDR) Waitlist	Acrophobia Symptoms Anxiety Sensitivity Index	6 sessions [∼60 min]	No	High	No	High
Bioulac et al. 2020	Reduce distractibility of Children with ADHD	Medication Psychotherapy	ADHD Rating Scale Virtual Classroom Task Continuous Performance Test	12 sessions in 6 weeks [30 min]	No	High	Low-quality	Low
Guldager et al. 2022	Enhance drinking and drug refusal self-efficacy	Active control (Oculus Quest – First Steps Application)	Drinking and Drug refusal skills Communication Skills regarding peer-pressure alcohol consumption	1 session [45 min]	No	Low	Low-quality	Moderate
Hadley et al. 2019	Teach sexual and substance use refusal skills	Emotion Regulation and Roleplay	HIV Prevention Knowledge and Self-Efficacy Diverse Emotion Regulation Scale	4 sessions in 4weeks [2 hours]	No	High	Low-quality	Moderate
Kahlon et al. 2023	Treat Public Speaking Anxiety	Waitlist & Online Psychoeducation Online Psychoeducation & Online Exposure	Public Speaking Anxiety Social Interaction Anxiety Social Phobia Eating Disorder Inventory	15 sessions in 6 weeks	Yes	Low	Low-quality	Low
Porras-Garcia et al. 2020	Treat anorexia nervosa	CBT	Eating Disorder Inventory Body Appreciation Scale Visual analog Scales	5 sessions in 5 weeks [∼1 hour]	No	Low	High-quality	Moderate
Tabrizi et al. 2020	Improve the memory of students with ADHD	Medication No Intervention	Memory Variable	10 sessions [3 min]	No	Low	No	Low
Wong et al. 2024	Improve social interaction skills of children with ADHD	Traditional social skills training Waitlist	Social Skills Improvement System Behavior Rating Inventory of Executive Function	12 sessions in 12 weeks [up to 20 min]	No	High	Low-quality	High

#### 3.2.1 Acrophobia (Azimisefat et al., 2022)

This VRET intervention was designed for female students, 16–18 years old, who met DSM-5 criteria for acrophobia. In total, there were four different scenarios to treat acrophobia. The sessions followed the standard protocols of CBT for specific phobias (Scozzari and Gamberini, 2011). The four different scenarios were walking up a high hill to its summit (1), walking on the roof of a building (2), climbing a ladder (3), and riding a balloon (4).

#### 3.2.2 ADHD (Bioulac et al., 2020, Tabrizi et al., 2020)

Bioulac et al. (2020) recruited children with ADHD, aged between seven and eleven years, for their study. They aimed to improve distractibility in their participants. For this they adapted a virtual classroom task for French-speaking patients (Bioulac et al., 2012). In this virtual classroom, a virtual teacher provided information and tasks to the children. At the same time, distractions were presented in the classroom (e.g., pencils dropping, paper airplanes flying, a car rumbling outside the windows).

The study of Tabrizi et al. (2020) has many similarities. They also recruited children with ADHD and used a virtual classroom. As Bioulac et al. (2020) the students also needed to perform tasks while audio-visual distractions were presented. In this, the students performed tasks while distractions were presented.

#### 3.2.3 Aggressive behavior (Alsem et al., 2023)

This intervention was designed to reduce aggressive behavior problems in boys. The boy’s age range was 8–13 years, and to participate, they must be referred for displaying aggressive behavior problems. The VR intervention was an implementation of the manualized CBT program *YourSkills*, which is based on evidence-based treatment for children with aggressive behavior problems (Lochman et al., 2008, van Manen, 2001). The intervention focused on children but also provided parent involvement.

In the VR intervention, the children could move freely within a 3x3 meter area, interact with virtual children and adults, and play various games. The therapists could manipulate the virtual situation and take over the virtual characters in real-time to evoke aggressive behavior.

#### 3.2.4 Anorexia nervosa (Porras-Garcia, 2020)

The intervention targeted female adolescents between 14 and 18 years old with anorexia nervosa. The aim was to reduce eating disorder symptoms, such as body dissatisfaction, physical appearance state anxiety, and fear of gaining weight. To conduct the intervention, the authors designed a virtual environment featuring a room with a large mirror in front of the participants. The participant embodied a young female avatar based on photos of the participants. Eye and body tracking were used to provide full-body motion tracking and measure the fixation time of the gaze towards weight-related body parts. Throughout the different sessions, the BMI of the virtual avatar was gradually increased until it reached a healthy BMI, starting from the same BMI as the participants. During the session, the participants were asked to focus on the virtual avatar’s body and think about their level of anxiety. The session ended when their anxiety level decreased.

#### 3.2.5 Risk behavior self-efficacy (Guldager et al., 2022, Hadley et al., 2019)

The VR intervention by Guldager et al. (2022) aimed to enhance the drinking refusal self-efficacy of students aged 15 to 18. The students did not need to meet any diagnostic criteria.

In the virtual environment, students were confronted with several behavioral options, where they were pressured and encouraged by (virtual) peers, in a party setting, to drink alcohol. They received visual feedback on their blood alcohol concentration based on their choices.

Hadley et al. (2019) focused on a similar target population, to improve emotion regulation skills and sexual and substance risk behavior for adolescents aged 12 to 15 years. They used four VR scenarios: adolescent party (1), condom purchasing (2), sexual negotiation (3), and HIV/STD testing (4).

The VR intervention was designed based on similar face-to-face group interventions by Brown et al., 2012, Houck et al., 2016. The participants were able to respond verbally to the virtual avatars’ actions and statements. The facilitators were able to immediately interact with the participant by selecting responses from a menu.

#### 3.2.6 Social skills (Kahlon et al., 2024, Wong et al., 2024)

Both Kahlon et al. (2024) and Wong et al. (2024) investigated the use of VR for social skill related issues. Kahlon et al. (2024) focused on providing a VR training application for adolescents with Public Speaking Anxiety between 13 and 16 years. A VR classroom was developed to implement this intervention. This VR classroom contained 6 to 16 virtual avatars, with variations in task duration, audience reaction, and presentation type. For each task, they were given a rating based on their performance in terms of speaking loudly and maintaining eye contact with the audience, with a maximum of five stars. They unlocked the next level if they reached a sufficient score of at least four stars. The participants were expected to complete 15 different variations during the intervention period.

Wong et al. (2024) aimed to enhance the social interaction skills of children with ADHD aged six to twelve years. Three scenarios were designed to simulate real social situations (1) classroom and playground, (2) mass transit railway system, (3) supermarket and restaurant. The children could interact with virtual avatars, representing other persons. They got real-time feedback on their interactions to provide immediate correction of participants’ behaviors.

**Fig. 2:**
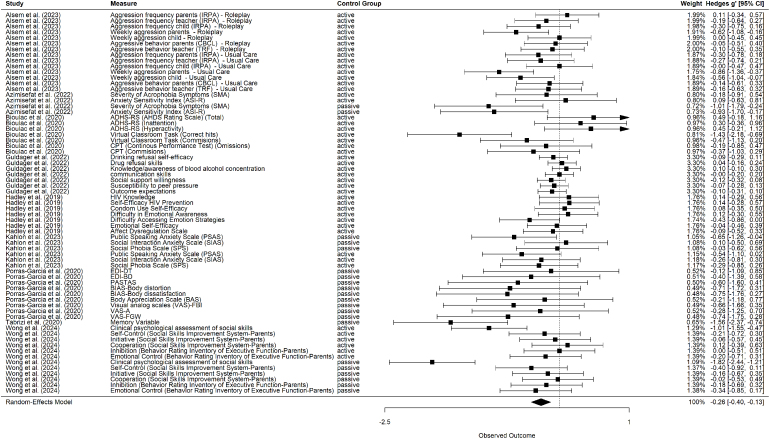
VR Interventions compared with active and passive control groups. Negative effect size = VR Intervention is more beneficial than the control group.

### 3.3 Control conditions

Five Studies (Azimisefat et al., 2022, Kahlon et al., 2024, Porras-Garcia, 2020, Tabrizi et al., 2020, Wong et al., 2024) compared a VR intervention with a passive control group (waitlist control, no treatment, etc.). One control group in a study was divided into two phases: a non-treatment phase and a self-study psychoeducation phase, each lasting three weeks (Kahlon et al., 2024). We found a second paper on the same study, measuring the outcomes after three weeks. We combined the results from both reports to better separate active and passive treatment (Kahlon et al., 2024, Kahlon et al., 2023). Seven studies (Alsem et al., 2023, Azimisefat et al., 2022, Bioulac et al., 2020, Guldager et al., 2022, Hadley et al., 2019, Kahlon et al., 2024, Wong et al., 2024) had at least one active control group (e.g., Roleplay, Psychotherapy, Traditional Social Skills Training).

### 3.4 Meta-analyses

**Fig. 3:**
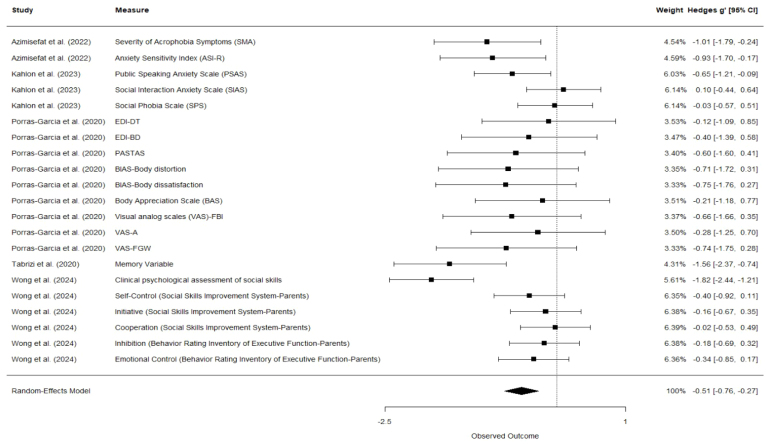
VR Interventions compared with passive control groups. Negative effect size = VR Intervention is more beneficial than the control group.

**Fig. 4:**
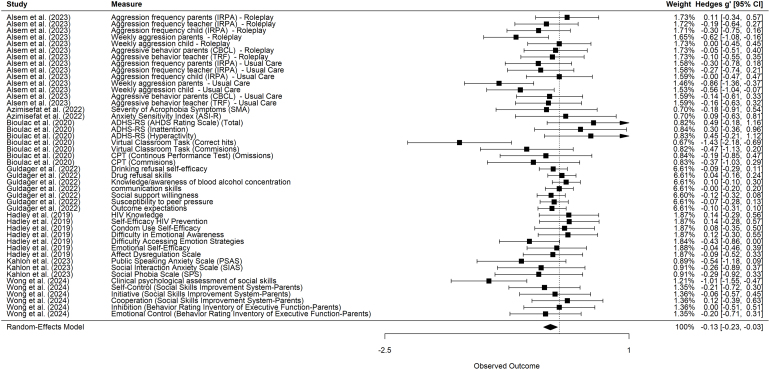
VR Interventions compared with active control groups. Negative effect size = VR Intervention is more beneficial than the control group.

The results of the VR intervention’s effects (compared with active and passive control conditions) on pre-post change scores are presented in Fig. 2. In the following, negative values of *g* indicate reduction of symptoms or improvement of skills. Positive values of *g* indicate a worsening of symptoms or a deterioration of skills. Fig. 3 presents a comparison of the effects of the VR intervention versus the passive control conditions. The comparison of the VR intervention effects with the active control conditions is reported in Fig. 4.

In Total, there are 51 unique measures across both passive and active control groups. Although some measures were reported for the active and passive control groups, a total of 67 measures and corresponding effect sizes were identified and calculated. Of these 67 effect sizes, 21 pertain to the passive control group and 46 to the active control group.

The effect of VR interventions compared to both, active and passive, control conditions was small and significant (*g* = −0.26, 95% CI [−0.40, −0.13], *p* < .01, Q = 133.23, $I^{2}=54.94\text{\%}$). Similarly, comparing the effect of VR interventions to active control conditions also revealed a small and significant effect (*g* = −0.13, 95% CI [−0.23, −0.03], *p* = .01, Q = 69.63, $I^{2}=20.42\text{\%}$). The comparison of VR interventions to passive control conditions revealed significant effects of moderate magnitude (*g* = −0.51, 95% CI [−0.76, −0.27], *p* < .01, Q = 43.71, $I^{2}=55.76\text{\%}$). The individual effect sizes range from −1.82 to 0.49. Then, comparing all control groups, 15 effect sizes are equal to 0 or positive, and 54 effect sizes are negative. Therefore, in 52 out of 67 cases, the VR intervention indicated a more beneficial effect on the patients’ mental health than the control group.

Regarding the passive control groups, 20 out of 21 effect sizes were negative, indicating a decrease in symptoms. Compared to the active control groups, 32 out of 46 effect sizes were negative.

A subgroup analysis for remote applications was not feasible, as only one Study (Kahlon et al., 2024) investigated a remote intervention.

To investigate whether the assumed value of r = 0.50 significantly influences our results, we conducted a sensitivity analysis by repeating the same three meta-analyses for values between r = 0.30 and r = 0.70. While there was a difference in the heterogeneity, Hedges *g* and the corresponding significance were not substantially affected by the results. The detailed values are accessible through the supplementary data.

### 3.5 Study quality

**Fig. 5:**
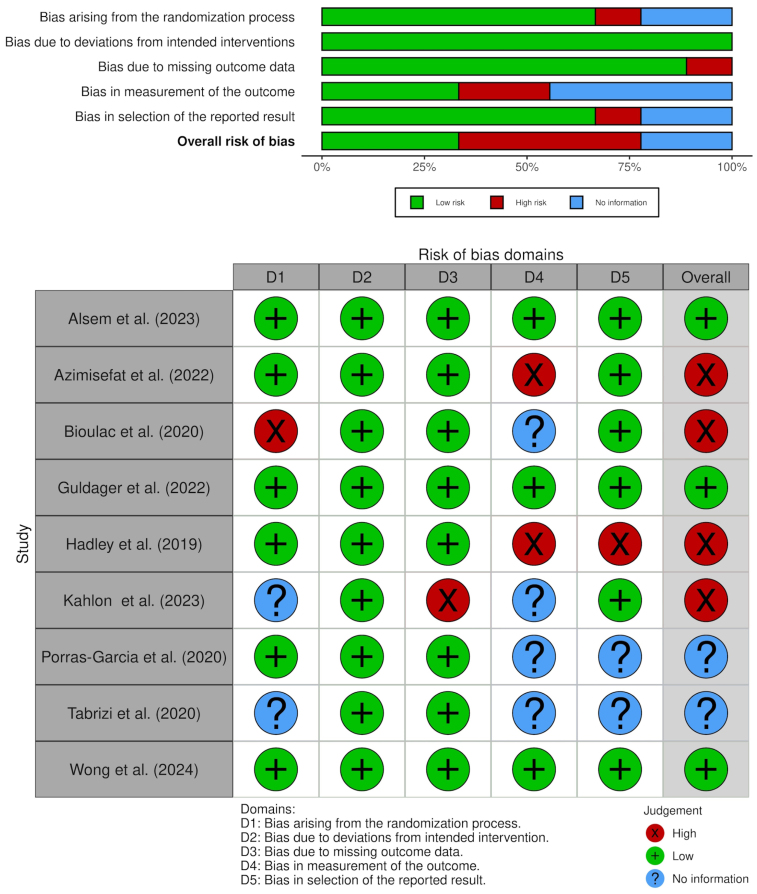
Cochrane Risk of Bias (ROB-2) - Summary and Traffic Lights.

The study quality was assessed according to Risk of Bias. The results of this assessment are shown in Fig. 5. Three studies (Alsem et al., 2023, Guldager et al., 2022, Wong et al., 2024) were evaluated as having low risk of bias. For four studies (Azimisefat et al., 2022, Bioulac et al., 2020, Hadley et al., 2019, Kahlon et al., 2024), the overall risk of bias is considered high, as at least one domain of bias was evaluated as high. For two studies (Porras-Garcia, 2020, Tabrizi et al., 2020), the evaluation of two domains of bias was impossible, due to insufficient information. Therefore, an overall judgement was not possible.

### 3.6 Explorative analyses

Because of the small number of studies and the heterogeneous intervention types and measures analyzed together, additional subgroup analyses were conducted to enable a more interpretable interpretation of the results.

One subgroup analysis was conducted to compare the preventive intervention (Hadley et al., 2019), Guldager et al. (2022) to the therapeutic interventions, including the remaining seven studies.

The preventive intervention subgroup analysis delivered results for the effect size: (*g* = −0.03, 95% CI [−0.11, −0.05], *p* = .40). For heterogeneity Q = 9.10, *p* = .77. Both tests were not significant.

In contrast, the sub group analysis of therapeutic interventions had a higher number of included outcomes (k = 53) and delivered significant results for both effect size (*g* = −0.33, 95% CI [−0.46, −0.20], *p* < .01) and heterogeneity (Q = 100.81, *p* < .01).

In terms of moderator analysis, for the overall analysis, the appearance of high-realistic avatars had a positive effect on the patients’ mental health measures (*z* = −0.49, *p* = .03). We also found that high interactivity (*z* = −0.25, *p* = .01) had a positive effect on the patients’ mental health measures. In terms of usability, high usability had a positive effect on mental health measures (*z* = −0.33, *p* < .01). These results should be interpreted cautiously, given the potential multiplicity arising from several tested moderators.

A further subgroup analysis was conducted for low Risk of Bias vs. High/Uncertain Risk of Bias studies. Low RoB results were (*g* = −0.20, 95% CI [−0.38, −0.02], p=0.31) and heterogeneity (Q = 71.24, *p* < .01). The subgroup analysis for High or Uncertain RoB studies delivered similar results (*g* = −0.36, 95% CI [−0.63, −0.09], *p* = .01) and heterogeneity (Q = 60.60, *p* < .01).

### 3.7 Publication bias

Publication bias can only be limitedly conducted and interpreted because no unpublished or incomplete studies could be found that reported sufficient data.

## 4 Discussion

We conducted a systematic review and meta-analysis of VR interventions and their efficacy in child and adolescent psychotherapy. Compared with prior reviews — which either focused on one disorder (e.g., ADHD, depression) or on costly CAVE systems — our work emphasizes readily accessible and affordable VR hardware suitable for individual practitioners and small clinics.

In total, nine studies were identified, comprising 67 effect sizes and involving a total of 929 children and adolescents. The overall pre-post change score, comparing VR intervention to both active and passive control groups, favored the VR intervention (*g* = −0.26). Regarding the comparison to passive control groups, mental health improvement was significantly higher (*g* = −0.51, with 20 out of 21 ES < 0). The mental health improvement compared to established active control groups was significantly lower and more ambiguous (*g* = −0.13, with 32 out of 46 effect sizes < 0).

All these studies were assessed due to Risk of Bias Analysis. Three studies had an overall very low bias, with all five bias domains being low. Four studies had at least one bias, which had to be assessed as high; therefore, there is some probability of an overall bias. For the two studies, some bias domains could not be assessed due to missing information. Therefore, although the other bias probability was judged to be low, the overall bias for the study must still be assessed as unknown.

Despite the differences between the included studies, such as different VR applications, control groups, treated mental health symptoms, and country, we found comparatively moderate heterogeneity in the results, ranging from 20.42% to 55.76% . Implicating that the results should be, at least partially, transferable to other similar treatments.

The conducted subgroup analyses revealed cues that realistic avatars, high usability, and a high level of interactivity may positively affect patients’ mental health.

The results of the meta-analyses are in line with the results of other systematic reviews for specific mental health conditions. Like (Valmaggia et al., 2016) and Freitas et al. (2021) investigated VR interventions, mainly for adults, discovered similar results for VR interventions compared to CBT and in vivo exposure therapy, we also found that VR interventions for children and adolescents are similar effective.

Some researchers assumed too high heterogeneity (Ridout et al., 2021, Wiebe et al., 2022) and therefore did not conduct a meta-analysis. Our results show that only moderate heterogeneity was found.

Our literature search revealed a major research gap for remote VR interventions, because only one study used it. Regarding VR remote psychotherapy applications, the literature consists mostly of prototypes and technical feasibility studies.

As assumed by the interviewed experts by Ong et al. (2024), highly realistic virtual avatars and interactive elements are important factors for successful VR interventions. The factor usability, which is crucial for establishing new therapeutic forms (von Below et al., 2023), could also be shown as important for treating patients’ mental health conditions.

To our knowledge, this is the first meta-analysis to focus on high-immersive, low-cost VR applications for the psychotherapy of children and adolescents. Hence, the results are characterized by a high ecological utility for the implementation of VR into clinical practice. In addition, we only included RCTs, resulting in a high methodological standard. We could also quantify the heterogeneity and show that it can be assessed as moderate. Our meta-analysis also reveals that VR applications can be as effective as standard therapy, such as CBT, EMDR, and Roleplays.

One limitation of our meta-analysis is that, despite the high number of compared effect sizes (k = 67), the comparatively small number of studies (N = 9) is notable. Maybe a search for studies in other languages than English and German would add additional studies. Another limitation is that a huge amount of the measured children and adolescents stems, with 378 out of 929 (41.4%), from one study (Guldager et al., 2022).

Another possible limitation is that four out of nine studies must be rated as potentially highly biased. Additionally, there was not enough information for two studies. Furthermore, the small number of studies (K=9) is insufficient for formal tests such as publication bias analysis.

Due to the small number of studies, significance may be over-interpreted. Additionally, the significance of the conducted subgroup analyses may be affected, such as the preventive intervention subgroup analysis (k=14 outcomes).

Furthermore, only one study was found using a remote intervention, therefore a subgroup analysis was not feasible.

Despite the heterogeneity of the study diagnoses and symptoms, multiple mental health conditions, due to the lack of studies, could not be investigated. Three studies treated symptoms of ADHD, two studies anxiety-related, three studies behavior-related, and one study eating disorder-related. However, no study investigated highly prevalent or relevant disorders in youth such as depression, OCD, autism spectrum disorder, psychosis, Post-traumatic stress disorder, or learning disorders. Because the included studies assessed multiple and clinically heterogeneous outcomes across different mental health conditions, this review provides a transdiagnostic overview of an innovative and still small research area, but necessarily limits clinical specificity. In particular, outcome- or disorder-specific effects may differ in magnitude, and effects that are robust for one condition could be attenuated or obscured when considered alongside weak or null effects in other domains. Accordingly, any pooled or cross-outcome summary should be interpreted cautiously and should not be taken to imply that the outcomes represent a single underlying clinical construct. Clinical implications are therefore presented as preliminary and descriptive, highlighting directions for future disorder-specific research rather than making condition-specific treatment recommendations.

This review offers many implications for future research and clinical practice. First, it emphasizes the need for further research in the emerging field of VR and AR technology in child and adolescent psychotherapy, particularly for affordable VR/AR devices. The rather low number of RCTs highlights this gap in research additionally. Secondly, future studies should further target a greater diversity of mental disorders, such as Depression, OCD, autism spectrum disorder, Psychosis, Post-traumatic stress disorder, and Learning disabilities.

Thirdly, best practice standards for designing and using VR interventions are outstanding. This encompasses all aspects of the intervention, from design and interactivity possibilities to the number of introduction sessions required for each age group.

Fourth, our study demonstrates that VR interventions can be successfully integrated into clinical practice, yielding a slightly better effect than most active control groups. It should be noted that, given the outcomes’ heterogeneity and different measure scale, clinical implications should be interpreted cautiously.

Lastly, only one VR intervention was conducted in a remote setting (Kahlon et al., 2024), despite the authors not describing the necessity of a therapist intervening during treatments. It is important to investigate this further, for example, whether the same application at home produces a similar effect size as in the clinic. This could also significantly impact clinical practice, as a remote VR intervention with a similar effect size to In-Person therapy would save therapists’ hours and lower the barriers, connected with psychotherapy (Cata and Hackbarth, 2014, Gu and Wang, 2022, Schaffler et al., 2022), therefore, enable better access for children and adolescents, especially in regions where therapists are scarce.

## 5 Conclusions

The meta-analysis shows that low-cost immersive VR applications are effective in child and adolescent psychotherapy. The improvement of mental health measures are especially high compared to passive control groups. Furthermore, the meta-analysis reveals that immersive VR interventions yield comparable, and in some cases even slightly better, therapeutic outcomes compared to those of traditional active control groups, such as cognitive-behavioral therapy (CBT) and social skills training. Important factors for effective VR interventions are the presence of virtual avatars, interactive Elements, and high usability.

Conducting further RCTs targeting diverse mental disorders in the future is needed to holistically investigate the effectiveness of VR intervention in the domain of children and adolescent psychotherapy. Further research in remote and blended settings could hugely improve clinical practice by alleviating the strain associated with a shortage of psychotherapists and multiple high barriers to accessing mental health counseling in many countries.

## CRediT authorship contribution statement

**Steffen Massanneck:** Mainly responsible for the Research Design, Literature search, Coding the literature for the meta-analysis, Conducting the meta-analysis, Writing of the original draft, Preparing manuscript for submission. **Lennart Seizer:** Conceptual help on the literature search and meta-analysis, Coding the literature for the meta-analysis, Reviewing the original draft. **Nadine N. Schmitt:** Conceptual help on the literature search, Coding the literature for the meta-analysis, Reviewing the original draft. **Anja Pascher:** Coding the literature for the meta-analysis. **Johanna Löchner:** Conceptual help on the research design, Reviewing the original draft.

## Declaration of competing interest

The authors declare that they have no known competing financial interests or personal relationships that could have appeared to influence the work reported in this paper.

## Appendix A Supplementary data

Supplementary data to this article can be found online at https://osf.io/9hmev/?view_only=6f9ba1abd55a41df864b6ebd52b9ead0.

## Appendix B B. search term

*(virtual reality OR vr OR augmented reality OR ar OR mixed reality OR merged reality OR extended reality OR xr OR oculus* OR Meta quest OR metaverse) AND (psychotherap* OR psycho therap* OR mental health OR intervention* OR psycholog* OR psychiatr* OR treatment*) AND (young adult OR teen* OR child* OR adolescent* OR pediatric* OR kids* OR infant* OR minor* OR juvenile* OR young people OR pubescent*) AND (randomized controlled trial* OR rct OR randomized controlled trial*).*
